# Targeting homologous recombination repair to potentiate fluoroquinolone efficacy in *Mycobacterium abscessus*

**DOI:** 10.1002/mlf2.70103

**Published:** 2026-09-22

**Authors:** Shuai Wang, Md Shah Alam, Buhari Yusuf, Jingran Zhang, Ziwen Lu, Xiaofan Zhang, H. M. Adnan Hameed, Mst. Sumaia Khatun, Chunyu Li, Lijie Li, Aleksey A. Vatlin, Aweke M. Belachew, Xirong Tian, Yamin Gao, Cuiting Fang, Rogers P. Zhang, Jun Li, Xinyue Wang, Liqiang Feng, Li Wan, Tianyu Zhang

**Affiliations:** ^1^ State Key Laboratory of Respiratory Disease, Guangzhou Institutes of Biomedicine and Health, Chinese Academy of Sciences Guangzhou China; ^2^ China‐New Zealand Joint Laboratory on Biomedicine and Health, Guangzhou Institutes of Biomedicine and Health, Chinese Academy of Sciences Guangzhou China; ^3^ Guangdong‐Hong Kong‐Macao Joint Laboratory of Respiratory Infectious Diseases, Guangzhou Institutes of Biomedicine and Health, Chinese Academy of Sciences Guangzhou China; ^4^ University of Chinese Academy of Sciences Beijing China; ^5^ School of Life Sciences University of Science and Technology of China Hefei China; ^6^ Institute of Environmental Engineering RUDN University Moscow Russia; ^7^ Guangzhou Medical University‐Guangzhou Institutes of Biomedicine and Health Joint School of Life Sciences, Guangzhou Medical University Guangzhou China; ^8^ Guangzhou National Laboratory Guangzhou China; ^9^ State Key Laboratory of Respiratory Disease, Guangzhou Chest Hospital Guangzhou China; ^10^ Shanghai Institute for Advanced Immunochemical Studies and School of Life Science and Technology ShanghaiTech University Shanghai China

## Abstract

Fluoroquinolones (FQs) are important for treating fast‐growing mycobacterial infections; however, their efficacy can be limited by intrinsic resistance and the emergence of acquired resistance. FQs induce lethal DNA double‐strand breaks (DSBs) by targeting topoisomerases. Through a transposon screen in *Mycobacterium abscessus*, we identified that disruption of *adnB*, a homologous recombination (HR) gene, markedly sensitized the pathogen to FQs. Subsequent study showed that deleting core HR components (*adnB*, *recO*, *recA*, *recR*, and *ruvB*) increased FQs' susceptibility, while other DSB repair pathways (single‐strand annealing or non‐homologous end joining) had no effect, demonstrating a unique reliance on HR for FQs' tolerance. Complementation with native or its homolog genes restored the resistance phenotype, indicating a conserved HR‐dependent tolerance mechanism in *M*. *abscessus*. In a murine model, genetic HR abrogation significantly improved FQs' therapeutic efficacy. These results highlight HR repair as a potential therapeutic target to potentiate FQs' activity and combat drug resistance in *M*. *abscessus* infections.

The emerging healthcare‐associated opportunistic pathogen *Mycobacterium abscessus* causes chronic pulmonary and soft‐tissue infections, especially in immunocompromised patients^1^. A significant increase in *M. abscessus* infections is being observed in East Asia, highlighting its emergence as a crucial pathogen among nontuberculous mycobacteria worldwide^2^. Its intrinsic multidrug resistance and increasing incidence create a formidable therapeutic challenge, highlighting the urgent need for new drug targets^1^, ^2^. Fluoroquinolones (FQs) are used to treat drug‐resistant tuberculosis and macrolide‐resistant *M. abscessus* ^3^, with moxifloxacin (MFX) showing potent *in vitro* activity^3^. FQs inhibit type II topoisomerases (gyrase or topoisomerase IV) principally for DNA replication and segregation, stabilizing cleavage complexes, preventing DNA re‐ligation, and causing lethal double‐strand breaks (DSBs) and bacterial cell death^4^.

Efficient repair of DSBs is crucial for maintaining genomic stability in mycobacteria. The repair of mycobacterial DSBs is mediated by three distinct routes: (i) homologous recombination (HR), a high‐fidelity RecA‐dependent mechanism that restores sequence integrity; (ii) non‐homologous end joining (NHEJ), an error‐prone Ku–LigD–dependent process that ligates broken DNA ends without homology; and (iii) single‐strand annealing (SSA), a RecA‐independent pathway requiring RecBCD and strand‐annealing proteins such as RecO, which often results in deletions between homologous repeats^5^. Additionally, in prokaryotes, HR is the predominant pathway for repairing DSBs among the three DSBs repair pathways^5^.

In this study, to investigate genetic determinants of intrinsic FQs' resistance in *M. abscessus*, we performed a transposon mutagenesis screen of >5000 mutants. One mutant, designated S27, showed markedly increased sensitivity to FQs (Figure 1A and Table S1). The minimum inhibitory concentrations (MICs) of two FQs, levofloxacin (LEV) and MFX, against strain S27 were reduced to 1/16 of those observed for wild‐type *M. abscessus* (WT) (Table S1). Sequencing revealed a transposon insertion within *MAB_3515c*, located 337 bp downstream of the start codon (Figure 1A). Sequence analysis showed that MAB_3515 shares 59.21% identity with *M. tuberculosis* AdnB, an HR DNA helicase, suggesting that it is the homolog (Figure S1).

**Figure 1:**
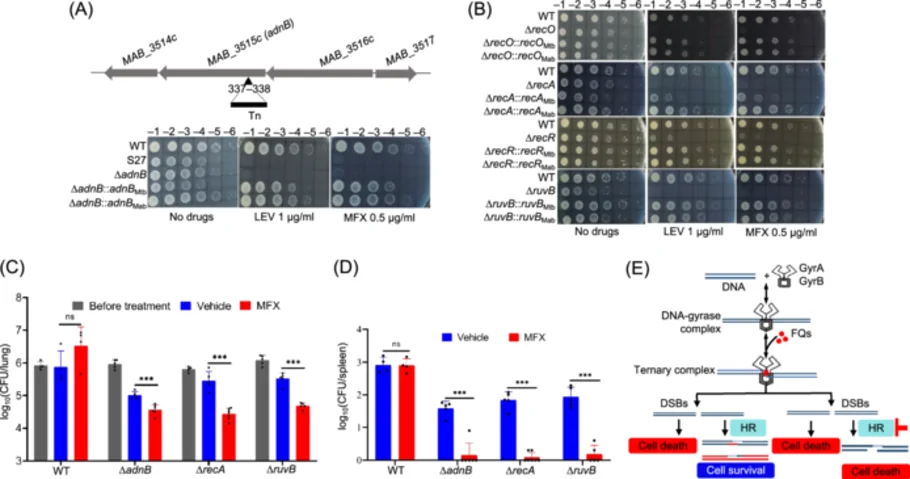
Suppression of HR enhances FQs' sensitivity in *Mycobacterium abscessus*. (A) Diagram showing the transposon insertion site within the *adnB* (*MAB_3515c*) gene and susceptibility of different *M. abscessus* strains to FQs (LEV and MFX). The insertion occurs at a “TA” dinucleotide, 337 bp downstream of the start codon. The numbers –1, –2, –3, –4, –5, and –6 indicate serial 10‐fold dilutions (i.e., 10⁻¹, 10⁻², 10⁻³, 10⁻⁴, 10⁻⁵, and 10⁻⁶). (B) Drug susceptibility of different *M. abscessus* strains to FQs. (C) *In vivo* evaluation of *M. abscessus* susceptibility to FQs. *M. abscessus* CFUs in the lungs of mice treated with MFX versus those treated with PBS (vehicle). (D) *M. abscessus* CFUs in the spleens of mice treated with MFX versus those treated with PBS (vehicle). Each data point represents an individual mouse. An unpaired *t*‐test was conducted for statistical significance. ns, no significant; ***p < 0.001. (E) Scheme describing synergistic FQs' sensitization by targeting the HR repair pathway. In wild‐type mycobacteria, a portion of FQs‐induced DSBs can be repaired by HR, allowing some cells to survive. However, when HR is disrupted, these DSBs remain unrepaired, rendering mycobacteria more sensitive to FQs. WT, wild‐type *M*. *abscessus*; S27, transposon (Tn) insertion site containing mutant; ∆*adnB*, *adnB* deletion strain; ∆*recO*, *recO* deletion strain; ∆*adnB*::*adnB*_Mab_, complemented strain expressing *adnB*_Mab_; and ∆*adnB*::*adnB*_Mtb_, complemented strain expressing *adnB*_Mtb_. Representative results from three independent experiments are shown. CFU, colony‐forming unit; DSBs, DNA double‐strand breaks; FQs, fluoroquinolones; HR, homologous recombination; LEV, levofloxacin; MFX, moxifloxacin; PBS, phosphate buffer saline.

To confirm the phenotype and exclude polar effects of transposon insertion, we constructed an in‐frame deletion of *adnB* in *M. abscessus* (∆*adnB*). Subsequently, we complemented it with the *adnB* gene derived from *M. abscessus* and *M. tuberculosis*, resulting in ∆*adnB*::*adnB*_Mab_ and *∆adnB::adnB*_Mtb_, respectively. Drug susceptibility testing revealed that the phenotype of ∆*adnB* was consistent with that of strain S27, showing hypersensitivity to LEV and MFX, while complementation with either *M. abscessus* or the *M. tuberculosis adnB* gene restored the WT resistance phenotype (Figure 1A and Table S1). Notably, deletion of *adnB* did not alter susceptibility to other antibiotics with unrelated mechanisms of action (Table S2), suggesting that *adnB* specifically modulates resistance to FQs in *M. abscessus*.

Given AdnB's evident functional role in HR and the increased FQs' sensitivity, we hypothesized that key HR components influence FQs' susceptibility in *M. abscessus*. HR repair in mycobacteria involves several coordinated steps: recognition of DSBs, 5′–3′ end resection to generate single‐stranded DNA, mediator‐assisted strand invasion into a homologous template, DNA synthesis, and resolution of Holliday junctions to restore genome integrity^6^. To assess the role of HR in mediating FQs' resistance, we constructed deletion mutants for key genes involved in distinct stages of HR—*recA*, *recO*, *recR*, and *ruvB*—as well as corresponding complementation strains. All HR‐deficient mutants showed pronounced FQs' hypersensitivity, with MICs reduced to 1/16–1/32 of WT (Figure 1B and Table S1). No changes were observed in susceptibility to drugs with unrelated mechanisms of action (Table S2), and growth had a minimal impact (Figure S2), indicating specific FQs' sensitization. Complementation with native or *M. tuberculosis* orthologs restored resistance, revealing functional conservation and confirming the essential role of these HR factors in maintaining FQs' resistance.

To explore whether HR disruption generally sensitizes *M. abscessus* to different DNA‐damaging agents, we tested the susceptibility of the same mutants to mitomycin C (MMC) and bleomycin (BLM). As shown in Table S1, HR‐deficient strains showed increased sensitivity to MMC and BLM (MICs reduced up to fourfold compared to WT), though the change was less pronounced than that observed with FQs. This differential effect may reflect the distinct nature of DNA lesions induced: While FQs predominantly generate DSBs, BLM primarily induces single‐strand breaks^7^, and MMC introduces interstrand cross‐links that obstruct replication forks^8^. Accordingly, the HR pathway plays a less prominent role in repairing damage caused by MMC and BLM compared to FQs‐induced DSBs.

In *M. abscessus*, DSBs are repaired not only through HR but also via SSA and NHEJ^6^. To further investigate the impact of SSA and NHEJ DSBs repair pathways on the sensitivity of *M. abscessus* to FQs, we constructed *recC* (SSA), *ku* (NHEJ), or *ligD (*NHEJ) knockout *M. abscessus* strains (∆*ku*, ∆*ligD*, and ∆*recC*) and assessed their sensitivity to FQs. In contrast to HR‐deficient mutants, the MICs for LEV and MFX against the *ku*, *ligD*, and *recC* knockout strains remained unchanged compared to the WT strain (16 and 8 μg/ml, respectively) (Table S3). These findings suggest that disrupting NHEJ and SSA does not affect the sensitivity of *M. abscessus* to FQs.

To validate the therapeutic potential of targeting HR repair enzymes, we evaluated the efficacy of MFX in a murine aerosol infection model using Δ*adnB*, Δ*recA*, and Δ*ruvB* strains (Figure 1C,D). In mice infected with WT, colony‐forming unit (CFU) counts in the lungs appeared to be slightly higher in the MFX‐treated group compared to the vehicle group, although the difference was not statistically significant (Figure 1C). In contrast, mice infected with HR‐deficient strains (∆*adnB*, ∆*recA*, and ∆*ruvB*) showed significant reduction in bacterial load upon MFX treatment, consistent with the *in vitro* findings. Among the knockout strains, *∆adnB* showed a slightly higher MIC to MFX compared to *∆recA* and ∆*ruvB*, which correlated with a relatively modest reduction in lung CFUs *in vivo* (Figure 1C). These data collectively indicate that disruption of the HR repair pathway sensitizes *M. abscessus* to FQs *in vivo*.

Interestingly, despite similar initial infection loads across all groups, mice infected with HR‐deficient strains showed significantly reduced lung CFUs by the end of the experiment, even without MFX treatment (*p* < 0.05), suggesting impaired bacterial persistence or virulence. In contrast, bacterial loads in the lungs of untreated mice infected with WT remained unchanged (*p* > 0.05) (Figure 1C). Consistently, in the untreated group, the bacterial burden of HR‐deficient strains in the spleen was lower than that of the WT (Figure 1D), further supporting the hypothesis and underscoring the dual impact of HR deficiency, both sensitizing bacteria to FQs and impairing their virulence.

Antibacterial synergy is a cornerstone of modern antimicrobial therapy. Rational combinations of antibiotics can significantly enhance therapeutic efficacy, limit the emergence of resistance, and potentially reduce drug‐related toxicity. Understanding and applying this concept are essential for optimizing clinical treatment strategies and improving patient outcomes. A classic example is the combination of β‐lactam antibiotics with β‐lactamase inhibitors^9^. Co‐administration of β‐lactamase inhibitors prevents enzymatic degradation, restoring antibiotic activity^9^. This synergistic approach not only improves antimicrobial efficacy but also reduces the likelihood of resistance development. In this context, our study identified a novel synergistic strategy, in which the disruption of the HR repair pathway markedly increased the sensitivity of mycobacteria to FQs. These data highlight targeting key HR enzymes to improve FQs' activity and enable synergistic combination therapeutic strategies.

The proposed mechanism by which HR pathway disruption sensitizes mycobacteria to FQs is illustrated in Figure 1E. During replication and other cellular processes, topoisomerases such as GyrA and GyrB generate transient DSBs to alleviate DNA supercoiling. FQs bind to the DNA–topoisomerase complex, forming a stabilized ternary structure that prevents re‐ligation of the DNA, ultimately resulting in bacterial cell death. In response to FQs‐induced DSBs, bacteria activate DNA repair mechanisms, with HR being the predominant high‐fidelity repair pathway in mycobacteria. HR‐mediated repair enables some bacterial cells to survive (Figure 1E, bottom left). However, in the absence of functional HR, DSBs cannot be efficiently repaired, leading to extensive bacterial death—even at sublethal concentrations of FQs (Figure 1E, bottom right). HR is a conserved and essential repair mechanism in many bacterial species. Given the broad‐spectrum activity of FQs against Gram‐positive and Gram‐negative bacteria, HR inhibition may represent a generalizable strategy to potentiate FQs' efficacy beyond mycobacteria. As previously reported, transposon sequencing in *Escherichia coli* revealed that ciprofloxacin exposure reduced insertions in HR genes (*recA*, *recB*, and *recC*)^10^. Similarly, *recA* deletion increases quinolone sensitivity in *Pseudomonas aeruginosa* and *Staphylococcus aureus* ^11^. In this study, no transposon insertions were found in other HR genes (*recA*, *recC*, *recO*, or *recR*), likely due to the limited quantity of the mutant library analyzed. These observations confirm that targeting HR or exploiting HR defects potentiates FQs' bactericidal activity across multiple bacterial species, suggesting broad therapeutic potential.

Additionally, our *in vivo* data demonstrate that disruption of the HR repair pathway significantly impairs the survival of mycobacteria, consistent with prior studies indicating that DNA repair deficiencies impair mycobacterial proliferation under macrophage‐simulated stress^12^. These findings suggest that targeting key components of the HR pathway could both augment susceptibility of mycobacteria to FQs and independently undermine their persistence within hosts.

Importantly, FQ sensitization was specific to HR disruption. This specificity is consistent with the central role of HR as the predominant DSBs repair pathway in mycobacteria^5^. HR, mediated by RecA and the AdnAB complex, enables high‐fidelity repair of DSBs using a homologous DNA template, which is predominantly available during DNA replication. Notably, FQs exert their bactericidal activity preferentially during this replication phase, thereby coupling DNA damage with replication‐dependent repair processes and underscoring the importance of HR in maintaining genomic stability under FQs‐induced stress^6^. In contrast, SSA and NHEJ function mainly as backup repair pathways and contribute minimally to the repair of FQs‐induced DSBs when genomic DNA is actively replicating^5^. Supporting this view, previous studies have reported that NHEJ shows limited capacity to resolve extensive DNA damage in *M. abscessus*, *M. tuberculosis*, and *M. smegmatis*, as CRISPR‐Cas–induced DSBs cannot be efficiently repaired unless the *nrgA* gene from *M. marinum* is ectopically expressed^13^, ^14^_._ These observations indicate that, under conditions of severe and persistent DSB stress, the endogenous NHEJ machinery alone is insufficient to ensure efficient DNA repair in these mycobacteria.

Prokaryotic and eukaryotic HR repair mechanisms differ significantly. Prokaryotes use RecA for homologous pairing in a relatively simple process involving fewer factors^15^. Eukaryotes use a complex network including RAD51, BRCA1, and BRCA2^16^. Though functionally analogous, RecA and RAD51 show structural and biochemical differences despite sharing a similar ATP‐binding domain^17^. Concerns that inhibiting HR might force error‐prone repair pathways like NHEJ or SSA, increasing mutagenesis, are mitigated in mycobacteria. Studies show an asymmetric relationship: Loss of NHEJ or SSA can be compensated by HR, but HR inactivation does not upregulate these mutagenic alternatives^5^, ^14^. Mycobacteria preferentially maintain high‐fidelity repair. These distinctions suggest that bacterial RecA inhibitors could specifically enhance FQs' efficacy without promoting adaptive evolution.

As previously reported, several compounds inhibit HR repair by targeting RecA^18^, ^19^. For example, phthalocyanine tetrasulfonic acid blocks RecA activity, enhancing quinolone efficacy against Gram‐positive and Gram‐negative bacteria, including in mouse models of *E. coli* infection^18^. In mycobacteria, particularly the *M. tuberculosis* complex, RecA contains an intein and requires protein splicing for activation^20^. Notably, Cisplatin, an FDA‐approved drug, inhibits this splicing, impairing RecA maturation and function^19^. These findings support developing RecA‐targeted adjuvants to co‐administer with FQs for treating drug‐resistant mycobacterial infections.

In conclusion, our work establishes HR as a key determinant of FQs' tolerance in *M. abscessus* and highlights HR‐mediated DNA repair as a promising therapeutic target. Targeting HR may represent a feasible strategy to potentiate FQs' activity and overcome intrinsic drug resistance in this highly drug‐resistant pathogen.

## ETHICS STATEMENT

Animal procedures were approved by the institutional animal care and use committee of the Guangzhou Institutes of Biomedicine and Health (No. 2024003).

## Supporting information

Supporting File 1.

Supporting File 2.

## Data Availability

The data that support the findings of this study are available from the corresponding authors upon request.

## References

[1] Olawoye IB , Waglechner N , McIntosh F , Akochy PM , Cloutier N , Grandjean Lapierre S , et al. Genomic epidemiology of *Mycobacterium abscessus* on the island of montreal is not suggestive of health care‐associated person‐to‐person transmission. J Infect Dis. 2025;231:e396–e406. 10.1093/infdis/jiae407 PMC11841644 39189818

[2] Hongmei S , Ruixia L , Jiankang L , Han W , Chang Z , Yan MY , et al. Prevalence of nontuberculous mycobacteria and the emergence of rare species in Henan province, China. Epidemiol Infect. 2024;152:e92. 10.1017/S095026882400061X PMC11748010 38708766

[3] Nie WJ , Xie ZY , Gao S , Teng TL , Zhou WQ , Shang YY , et al. Efficacy of moxifloxacin against *Mycobacterium abscessus* in zebrafish model *in vivo* . Biomed Environ Sci. 2020;33:350–358. 10.3967/bes2020.047 32553079

[4] Collin F , Karkare S , Maxwell A . Exploiting bacterial DNA gyrase as a drug target: current state and perspectives. Appl Microbiol Biotechnol. 2011;92:479–497. 10.1007/s00253-011-3557-z PMC3189412 21904817

[5] Gupta R , Barkan D , Redelman‐Sidi G , Shuman S , Glickman MS . Mycobacteria exploit three genetically distinct DNA double‐strand break repair pathways. Mol Microbiol. 2011;79:316–330. 10.1111/j.1365-2958.2010.07463.x PMC3812669 21219454

[6] Glickman MS . Double‐strand DNA break repair in mycobacteria. Microbiol Spectr. 2014;2: 10.1128/microbiolspec.MGM2-0024-2013. 26104351

[7] Chen J , Ghorai MK , Kenney G , Stubbe J . Mechanistic studies on bleomycin‐mediated DNA damage: multiple binding modes can result in double‐stranded DNA cleavage. Nucleic Acids Res. 2008;36:3781–3790. 10.1093/nar/gkn302 PMC2441780 18492718

[8] Bizanek R , McGuinness BF , Nakanishi K , Tomasz M . Isolation and structure of an intrastrand cross‐link adduct of mitomycin C and DNA. Biochemistry. 1992;31:3084–3091. 10.1021/bi00127a008 1554696

[9] Bush K , Bradford PA . β‐Lactams and β‐lactamase inhibitors: an overview. Cold Spring Harb Perspect Med. 2016;6:a025247. 10.1101/cshperspect.a025247 PMC4968164 27329032

[10] Tran T , Ran Q , Ostrer L , Khodursky A . *De novo* characterization of genes that contribute to high‐level ciprofloxacin resistance in *Escherichia coli* . Antimicrob Agents Chemother. 2016;60:6353–6355. 10.1128/AAC.00889-16 PMC5038283 27431218

[11] Mercolino J , Lo Sciuto A , Spinnato MC , Rampioni G , Imperi F . RecA and specialized error‐prone DNA polymerases are not required for mutagenesis and antibiotic resistance induced by fluoroquinolones in *Pseudomonas aeruginosa* . Antibiotics. 2022;11:325. 10.3390/antibiotics11030325 PMC8944484 35326787

[12] Kurthkoti K , Varshney U . Distinct mechanisms of DNA repair in mycobacteria and their implications in attenuation of the pathogen growth. Mech Ageing Dev. 2012;133:138–146. 10.1016/j.mad.2011.09.003 21982925

[13] Yan MY , Li SS , Ding XY , Guo XP , Jin Q , Sun YC . A CRISPR‐assisted nonhomologous end‐joining strategy for efficient genome editing in *Mycobacterium tuberculosis* . mBio. 2020;11:e02364–19. 10.1128/mBio.02364-19 PMC6989103 31992616

[14] Zeng S , Ju Y , Alam MS , Lu Z , Hameed HMA , Li L , et al. A CRISPR‐nonhomologous end‐joining‐based strategy for rapid and efficient gene disruption in *Mycobacterium abscessus* . mLife. 2025;4:169–180. 10.1002/mlf2.70007 PMC12042118 40313975

[15] Wright WD , Shah SS , Heyer WD . Homologous recombination and the repair of DNA double‐strand breaks. J Biol Chem. 2018;293:10524–10535. 10.1074/jbc.TM118.000372 PMC6036207 29599286

[16] Mirman Z , Sharma K , Carroll TS , de Lange T . Expression of BRCA1, BRCA2, RAD51, and other DSB repair factors is regulated by CRL4^WDR70^ . DNA Repair. 2022;113:103320. 10.1016/j.dnarep.2022.103320 PMC9474743 35316728

[17] Cox MM . Motoring along with the bacterial RecA protein. Nat Rev Mol Cell Biol. 2007;8:127–138. 10.1038/nrm2099 17228330

[18] Alam MK , Alhhazmi A , DeCoteau JF , Luo Y , Geyer CR . RecA inhibitors potentiate antibiotic activity and block evolution of antibiotic resistance. Cell Chem Biol. 2016;23:381–391. 10.1016/j.chembiol.2016.02.010 26991103

[19] Zhang L , Zheng Y , Callahan B , Belfort M , Liu Y . Cisplatin inhibits protein splicing, suggesting inteins as therapeutic targets in mycobacteria. J Biol Chem. 2011;286:1277–1282. 10.1074/jbc.M110.171124 PMC3020735 21059649

[20] Schneider RF , Hallstrom K , DeMott C , McDonough KA . Conditional protein splicing of the *Mycobacterium tuberculosis* RecA intein in its native host. Sci Rep. 2024;14:20664. 10.1038/s41598-024-71248-y PMC11377839 39237639

