# Supplementary material for: Targeting homologous recombination repair to potentiate fluoroquinolone efficacy in Mycobacterium abscessus
Source: mLife. 2026 Sep 22;5(5):670–4. doi: 10.1002/mlf2.70103 (PMC13595048; doi:10.1002/mlf2.70103)
Supplement: Supplementary file 1 [file MLF2-5-670-s001.docx]

**Targeting homologous recombination repair to potentiate fluoroquinolone efficacy in *Mycobacterium abscessus***

Shuai Wang ^1,2,3＃^, Md Shah Alam ^1,2,3,4＃^, Buhari Yusuf ^1,2,3,4^, Jingran Zhang ^1,2,3,5^, Ziwen Lu ^1,2,3,4^, Xiaofan Zhang ^1,2,3,4^, H.M. Adnan Hameed ^1,2,3^, Mst. Sumaia Khatun ^1,2,3,4^, Chunyu Li ^1,2,3,4^, Lijie Li ^1,2,3,5^, Aleksey A. Vatlin ^6^, Aweke M. Belachew ^1,2,3,4^, Xirong Tian ^1,2,3,7,10^, Yamin Gao ^1,2,3^, Cuiting Fang ^1,2,3,8,10^, Rogers Peng Zhang ^8^, Jun Li ^9^, Xinyue Wang ^8,10^, Liqiang Feng ^2,3,4^, Li Wan ^2,3,4^, Tianyu Zhang ^1,3,4,7,8,10*^

^1^ State Key Laboratory of Respiratory Disease, Guangzhou Institutes of Biomedicine and Health, Chinese Academy of Sciences, Guangzhou, China

^2^ China-New Zealand Joint Laboratory on Biomedicine and Health, Guangzhou Institutes of Biomedicine and Health, Chinese Academy of Sciences, Guangzhou, China

^3^ Guangdong-Hong Kong-Macao Joint Laboratory of Respiratory Infectious Diseases, Guangzhou Institutes of Biomedicine and Health, Chinese Academy of Sciences, Guangzhou, China

^4^ University of Chinese Academy of Sciences, Beijing, China

^5^ School of Life Sciences, University of Science and Technology of China, Hefei, China

^6^ Institute of Environmental Engineering, RUDN University, Moscow, Russia

^7^ Guangzhou Medical University-Guangzhou Institutes of Biomedicine and Health Joint School of Life Sciences, Guangzhou Medical University, Guangzhou, China

^8^ State Key Laboratory of Respiratory Disease, Guangzhou Chest Hospital, Guangzhou, China

^9^ Shanghai Institute for Advanced Immunochemical Studies and School of Life Science and Technology, Shanghai Tech University, Shanghai, China

^10^ Guangzhou National Laboratory, Guangzhou, China

^*^Correspondence: Tianyu Zhang, zhang_tianyu@gibh.ac.cn^zhang_tianyu@gibh.ac.cn^

^＃^ These authors contributed equally to this work.

**Materials and Methods**

**Bacterial strains, growth conditions, and antimicrobial agents**

*Escherichia coli* DH5α was cultured at 37℃ in Luria-Bertani (LB) broth and on LB agar plates. *Mycobacterium abscessus* subsp. *abscessus* GZ002 ^1^, *Mycobacterium tuberculosis* H37Ra, and their derivative strains were grown at 37℃ in Middlebrook 7H9 broth (Difco) supplemented with 10% oleic acid-albumin-dextrose-catalase (OADC, Difco) and 0.05% Tween 80, or on Middlebrook 7H10 agar (Difco) containing 10% OADC. Kanamycin and zeocin were used as needed at final concentrations of 100 μg/ml, and 30 μg/ml, respectively.

**Generation of fluoroquinolones-hypersensitive Tn mutants**

Transposon mutant libraries of *M. abscessus* were constructed using *Himar1* mutagenesis, as previously described ^1^. Briefly, 100 ml of mid-log-phase *M. abscessus* culture, with an optical density at 600 nm (OD_600_) of approximately 0.7 to 1.0, was infected with 1 ~ 2 × 10¹¹ PFU/ml of MycoMarT7 phage at 37℃ for 4 hours. Following infection, cells were pelleted, washed, and plated on Middlebrook 7H10 agar containing kanamycin. Tn mutants were then replica-streaked onto Middlebrook 7H10 agar with or without a subinhibitory concentration of levofloxacin (LEV) (2 μg/ml). Mutants showing growth impairment on LEV-containing plates were collected and subjected to minimum inhibitory concentration (MIC) determination. The Tn insertion sites in the LEV-hypersensitive mutants were identified using whole-genome sequencing and confirmed by PCR ^1^.

**Gene knockout and complementation**

Gene knockout in *M. abscessus* was performed using recombineering or CRISPR-assisted recombineering, following established protocols ^1^. For recombineering, an allelic exchange substrate for gene deletion was constructed by amplifying the upstream and downstream flanking arms and cloning them adjacent to a zeocin resistance gene, which was inserted into the pBluescript II SK (+) vector. These constructs were amplified by PCR and electroporated into freshly prepared electrocompetent *M. abscessus* cells carrying the recombineering plasmid pJV53. For CRISPR-assisted recombineering, the substrates, together with the crRNA-expressing pCRZeo plasmid, were electroporated into *M. abscessus* cells carrying the recombineering plasmid pJV53-Cpf1. Plates were incubated at 30℃ for 5 days. Colonies were selected on zeocin-containing plates and verified by PCR and sequencing. Subsequently, complemented strains were constructed by reintroducing targeted genes and their homolog from *M*. *tuberculosis*. All primers used in this study are listed in Table S4.

**Antibiotic susceptibility testing**

MICs were determined using the broth microdilution method. Briefly, mycobacterial cells were inoculated at a density of 5 × 10⁵ CFU/ml into 7H9 medium (without Tween 80) containing 2-fold serial dilutions of the drugs. *M. abscessus* strains were incubated at 37℃ for 14 days for clarithromycin and for 3 days for other drugs. The MIC was defined as the lowest antibiotic concentration that prevented visible bacterial growth. Herein, wild-type, knockout, and complement strains were propagated in 7H9 medium at 37℃ until reaching an OD_600_ of 0.6. To perform drug susceptibility, ten-fold serial dilutions were employed to both standard Middlebrook 7H10 agar (as a drug-free control) and media containing different concentrations of fluoroquinolones. Plates were incubated at 37°C for three days before the final reading. The experiment was performed in triplicate and repeated independently three times.

**Mouse infection and treatment**

Following previously established protocols ^1^, female BALB/c mice (GemPharmatech Co., Ltd, 8 weeks old) were infected with *M. abscessus* using a whole-body inhalation exposure system. To ensure appropriate immunosuppression, the mice were treated with dexamethasone (Sigma-Aldrich). Dexamethasone was dissolved in sterile 1 × phosphate-buffered saline (PBS, pH 7.4) and administered via daily subcutaneous injection at a dosage of 4 mg/kg/day, starting 1 week prior to infection and continuing throughout the experiment. Mice were sacrificed 4 hours post-infection to assess the initial bacterial burden in the lungs.

Subsequently, the mice received moxifloxacin at a dose of 100 mg/kg via oral gavage once daily, with PBS as the solvent. After 10 days of drug treatment, the mice were euthanized, and their lungs and spleens were aseptically removed, homogenized in PBS, and plated on Middlebrook 7H11 agar supplemented with 10% OADC. Colonies were counted after 3 days of incubation at 37℃. All animal care and experimental protocols were approved by the Institutional Animal Ethics Committee of the Guangzhou Institutes of Biomedicine and Health, Chinese Academy of Sciences.

**References**

1. Wang S, Cai X, Yu W, Zeng S, Zhang J, Guo L, et al. Arabinosyltransferase C mediates multiple drugs intrinsic resistance by altering cell envelope permeability in *Mycobacterium abscessus*. *Microbiol Spectr.* 2022; 10: e0276321.
