# Supplementary material for: Targeting homologous recombination repair to potentiate fluoroquinolone efficacy in Mycobacterium abscessus
Source: mLife. 2026 Sep 22;5(5):670–4. doi: 10.1002/mlf2.70103 (PMC13595048; doi:10.1002/mlf2.70103)
Supplement: Supplementary file 2 [file MLF2-5-670-s002.docx]

**Targeting homologous recombination repair to potentiate fluoroquinolone efficacy in *Mycobacterium abscessus***

Shuai Wang ^1,2,3＃^, Md Shah Alam ^1,2,3,4＃^, Buhari Yusuf ^1,2,3,4^, Jingran Zhang ^1,2,3,5^, Ziwen Lu ^1,2,3,4^, Xiaofan Zhang ^1,2,3,4^, H.M. Adnan Hameed ^1,2,3^, Mst. Sumaia Khatun ^1,2,3,4^, Chunyu Li ^1,2,3,4^, Lijie Li ^1,2,3,5^, Aleksey A. Vatlin ^6^, Aweke M. Belachew ^1,2,3,4^, Xirong Tian ^1,2,3,7,10^, Yamin Gao ^1,2,3^, Cuiting Fang ^1,2,3,8,10^, Rogers Peng Zhang ^8^, Jun Li ^9^, Xinyue Wang ^8,10^, Liqiang Feng ^2,3,4^, Li Wan ^2,3,4^, Tianyu Zhang ^1,3,4,7,8,10*^

^1^ State Key Laboratory of Respiratory Disease, Guangzhou Institutes of Biomedicine and Health, Chinese Academy of Sciences, Guangzhou, China

^2^ China-New Zealand Joint Laboratory on Biomedicine and Health, Guangzhou Institutes of Biomedicine and Health, Chinese Academy of Sciences, Guangzhou, China

^3^ Guangdong-Hong Kong-Macao Joint Laboratory of Respiratory Infectious Diseases, Guangzhou Institutes of Biomedicine and Health, Chinese Academy of Sciences, Guangzhou, China

^4^ University of Chinese Academy of Sciences, Beijing, China

^5^ School of Life Sciences, University of Science and Technology of China, Hefei, China

^6^ Institute of Environmental Engineering, RUDN University, Moscow, Russia

^7^ Guangzhou Medical University-Guangzhou Institutes of Biomedicine and Health Joint School of Life Sciences, Guangzhou Medical University, Guangzhou, China

^8^ State Key Laboratory of Respiratory Disease, Guangzhou Chest Hospital, Guangzhou, China

^9^ Shanghai Institute for Advanced Immunochemical Studies and School of Life Science and Technology, Shanghai Tech University, Shanghai, China

^10^ Guangzhou National Laboratory, Guangzhou, China

^*^Correspondence: Tianyu Zhang, zhang_tianyu@gibh.ac.cnzhang_tianyu@gibh.ac.cn

^＃^ These authors contributed equally to this work.

**Figure S1:** The alignment of amino acid sequences of AdnB proteins from *Mycobacterium abscessus* (MAB_3515) and *Mycobacterium tuberculosis*. *M. abscessus* showed 59.21% identity with AdnB of *M. tuberculosis*.

**Figure S2:** Growth curves of different *Mycobacterium abscessus* strains. Growth curves were measured by OD₆₀₀ for the deletion mutants of (A) *adnB*, (B) *recO*, (C) *recA*, (D) *recR*, and (E) *ruvB* and the corresponding complementary strains with the wild-type *M. abscessus* (WT) as a control under standard culture conditions. Values are means ± SD.

**Table S1** MICs of various drugs against different *M. abscessus* strains

| Strains^a^ | HR repair process | | Drugs/MICs^b^ (µg/ml) | | | |
| --- | --- | --- | --- | --- | --- | --- |
|  |  |  | LEV | MFX | MMC | BLM |
| WT | / | | 16 | 8 | 4 | 1 |
| S27 | / | | 1 | 0.5 | 1 | 0.5 |
| ∆*adnB* | | End resection helicase-nuclease | 1 | 0.5 | 1 | 0.5 |
| ∆*adnB*::*adnB*_Mab_ | |  | 8 | 4 | 4 | 1 |
| ∆*adnB*::*adnB*_Mtb_ | |  | 4 | 2 | 2 | 1 |
| ∆*recO* | | Resecting the ends to form single-stranded DNA | 1 | 0.25 | 1 | 1 |
| ∆*recO*::*recO*_Mab_ | |  | 16 | 8 | 4 | 1 |
| ∆*recO*::*recO*_Mtb_ | |  | 16 | 8 | 2 | 1 |
| ∆*recA* | Facilitating strand invasion | | 1 | 0.25 | 1 | 0.5 |
| ∆*recA*::*recA*_Mab_ |  |  | 16 | 8 | 4 | 1 |
| ∆*recA*::*recA*_Mtb_ |  |  | 16 | 8 | 2 | 1 |
| ∆*recR* | Essential for HR-mediator activity for RecA loading | | 1 | 0.5 | 1 | 0.5 |
| ∆*recR*::*recR*_Mab_ |  |  | 16 | 8 | 4 | 1 |
| ∆*recR*::*recR*_Mtb_ |  |  | 16 | 8 | 2 | 1 |
| ∆*ruvB* | Resolving the resulting Holliday junctions to restore genomic integrity | | 1 | 0.25 | 1 | 0.5 |
| ∆*ruvB*::*ruvB*_Mab_ |  |  | 16 | 8 | 4 | 1 |
| ∆*ruvB*::*ruvB*_Mtb_ |  |  | 16 | 8 | 2 | 1 |
| ^a^WT, wild-type *M*. *abscessus*; S27, Mab strain with transposon (Tn) insertion in *adnB*; ∆*adnB*, *adnB* deletion strain; ∆*recO*, *recO* deletion strain; ∆*adnB*::*adnB*_Mab_, complemented strain expressing *adnB*_Mab_; ∆*adnB*::*adnB*_Mtb_, complemented strain expressing *adnB*_Mtb_.  ^b^MIC is defined as the lowest concentration of a drug that inhibits visible bacterial growth.  LEV, levofloxacin; MFX, moxifloxacin; MMC, mitomycin C; BLM, bleomycin. | | | | | | |

**Table S2** MICs of drugs for different *M*. *abscessus* strains

| Strains | Drugs^a^/MICs^b^ (µg/ml) | | | | | | | | |
| --- | --- | --- | --- | --- | --- | --- | --- | --- | --- |
|  | RIF | IMP | CLA | LZD | TIG | RFB | AMK | VAN | CLF |
| WT | 128 | 16-32 | 64 | 64 | 2 | 8 | 8 | 64-128 | 2-4 |
| S27 | 128 | 16-32 | 64 | 64 | 2 | 8 | 8 | 64-128 | 2-4 |
| ∆*adnB* | 128 | 16-32 | 64 | 64 | 2 | 8 | 8 | 64-128 | 2-4 |
| ∆*adnB*::*adnB*_Mab_ | 128 | 16-32 | 64 | 64 | 2 | 8 | 8 | 64-128 | 2-4 |
| ∆*adnB*::*adnB*_Mtb_ | 128 | 16-32 | 64 | 64 | 2 | 8 | 8 | 64-128 | 2-4 |
| ∆*recO* | 128 | 16-32 | 64 | 64 | 2 | 8 | 8 | 64-128 | 2-4 |
| ∆*recO*::*recO*_Mab_ | 128 | 16-32 | 64 | 64 | 2 | 8 | 8 | 64-128 | 2-4 |
| ∆*recO*::*recO*_Mtb_ | 128 | 16-32 | 64 | 64 | 2 | 8 | 8 | 64-128 | 2-4 |
| ∆*recA* | 128 | 16-32 | 64 | 64 | 2 | 8 | 8 | 64-128 | 2-4 |
| ∆*recA*::*recA*_Mab_ | 128 | 16-32 | 64 | 64 | 2 | 8 | 8 | 64-128 | 2-4 |
| ∆*recA*::*recA*_Mtb_ | 128 | 16-32 | 64 | 64 | 2 | 8 | 8 | 64-128 | 2-4 |
| ∆*recR* | 128 | 16-32 | 64 | 64 | 2 | 8 | 8 | 64-128 | 2-4 |
| ∆*recR*::*recR*_Mab_ | 128 | 16-32 | 64 | 64 | 2 | 8 | 8 | 64-128 | 2-4 |
| ∆*recR*::*recR*_Mtb_ | 128 | 16-32 | 64 | 64 | 2 | 8 | 8 | 64-128 | 2-4 |
| ∆*ruvB* | 128 | 16-32 | 64 | 64 | 2 | 8 | 8 | 64-128 | 2-4 |
| ∆*ruvB*::*ruvB*_Mab_ | 128 | 16-32 | 64 | 64 | 2 | 8 | 8 | 64-128 | 2-4 |
| ∆*ruvB*::*ruvB*_Mtb_ | 128 | 16-32 | 64 | 64 | 2 | 8 | 8 | 64-128 | 2-4 |
| ^a^The clarithromycin susceptibility results were recorded at 14 days due to its inducible resistance characteristics, while the results for other antibiotics were recorded at 3 days.  ^b^MIC is defined as the lowest concentration of a drug that inhibits visible bacterial growth.  WT, wild-type *M*. *abscessus*; S27, Mab strain with transposon (Tn) insertion in *adnB*; ∆*recA*, *recA* deletion strain; ∆*recO*, *recO* deletion strain; ∆*recA*::*recA*_Mab_, complemented strain expressing *recA*_Mab_; ∆*recA*::*recA*_Mtb_, complemented strain expressing *recA*_Mtb_.  RIF, rifampicin; IMP, imipenem; CLA, clarithromycin; LZD, linezolid; TIG, tigecycline; RFB, rifabutin; AMK, amikacin; VAN, vancomycin; CLF, clofazimine. | | | | | | | | | |

**Table S3** MICs of antibiotics for different *M*. *abscessus* strains

| Antibiotics^a^ | Strains/ MICs^b^ (μg/ml) | | | |
| --- | --- | --- | --- | --- |
|  | WT | Δ*ku* | Δ*ligD* | Δ*recC* |
| LEV | 16 | 16 | 16 | 16 |
| MFX | 8 | 8 | 8 | 8 |
| BLM | 1 | 1 | 1 | 1 |
| MMC | 4 | 4 | 4 | 4 |
| RFB | 8 | 8 | 8 | 8 |
| TIG | 2 | 2 | 2 | 2 |
| AMK | 8 | 8 | 8 | 8 |
| CLF | 2-4 | 2-4 | 2-4 | 2-4 |
| IMP | 16-32 | 16-32 | 16-32 | 16-32 |
| VAN | 64-128 | 64-128 | 64-128 | 64-128 |
| CLA | 64 | 64 | 64 | 64 |
| RIF | 128 | 128 | 128 | 128 |
| LZD | 64 | 64 | 64 | 64 |
| ^a^The clarithromycin susceptibility results were recorded at 14 days due to its inducible resistance characteristics, while the results for other antibiotics were recorded at 3 days.  ^b^MIC is defined as the lowest concentration of a drug that inhibits visible bacterial growth.  LEV, levofloxacin; MFX, moxifloxacin; BLM, bleomycin; MMC, mitomycin C; RFB, rifabutin; TIG, tigecycline; AMK, amikacin; CLF, clofazimine; IMP, imipenem; VAN, vancomycin; CLA, clarithromycin; RIF, rifampicin; LZD, linezolid. | | | | |

**Table S4** Primers used in this study

| Primers | Nucleotide sequences (5’ – 3’) | Description |
| --- | --- | --- |
| adnBUP-F | CGCTCTAGAACTAGTGGATCCAAAGCCCTGCCACCAAAGA | Construction of the pBluescript II SK (+)-UadnBD plasmid containing the upstream and downstream homologous arms of *M. abscessus adnB* |
| adnBUP-R | TAGCCGTCGCAAGCTTGGAAGGCCAGCTGCCATAA |  |
| adnBDown-F | TTCCAAGCTTGCGACGGCTACGGAAGTTT |  |
| adnBDown-R | CTCGAGGTCGACGGTATCGATAGCGGCGTGATCACAAGGA |  |
| HindIIIdifzeo-F^a^ | CCCAAGCTTAACGCCGATAAGACACATTATGTCAGTTGAATTCCGACCCGCACGAC | PCR amplification of zeocin resistance cassette with *dif* sites for constructing homologous recombination substrates |
| HindIIIdifzeo-R^a^ | CCCAAGCTTAACTGACATAATGTGTCTTATCGGCGTTTCAGTCCTGCTCCTCGGCCA |  |
| adnBUZD-F | ATGGGGTGATATCGCCATAG | Amplification of homologous recombination substrates for construction of the *adnB* knockout mutant in *M. abscessus* |
| adnBUZD-R | AAAGCCCTGCCACCAAAGA |  |
| czadnB-F | GGCCAAGACAATTGCGGATCCGTCAGTCGTGAATGCATAC | Construction of a complementation plasmid for ∆adnBCMab |
| czadnB-R | GTTAACTACGTCGACATCGATACCAGGACAATCAGGAACA |  |
| recOUP-F | CTCGAGGTCGACGGTATCGATCAGCTCGCAGGTGTTGGTCT | Construction of the pBluescript II SK (+)-UrecOD plasmid containing the upstream and downstream homologous arms of *M. abscessus recO* |
| recOUP-R | TAGCTTGCGTTGACGGGTCAGCAGGGTCACG |  |
| recODown-F | TGACCCGTCAACGCAAGCTACGGACACTC |  |
| recODown-R | CGCTCTAGAACTAGTGGATCCGTAGACGCTGCTCCACAACC |  |
| recAUP-F | CGCTCTAGAACTAGTGGATCCTGGTGGGGGACACGGCTTT | Construction of the pBluescript II SK (+)-UrecAD plasmid containing the upstream and downstream homologous arms of *M. abscessus recA* |
| recAUP-R | CATATCGCCGGTCGGGATCACGGAGAT |  |
| recADown-F | TGATCCCGACCGGCGATATGGGCGTGGAGCAG |  |
| recADown-R | CTCGAGGTCGACGGTATCGATCCGAGGCGAGGGCATTCTT |  |
| ruvBUP-F | CGCTCTAGAACTAGTGGATCCGCAGAGGAACAACGGAAGG | Construction of the pBluescript II SK (+)-UruvBD plasmid containing the upstream and downstream homologous arms of *M. abscessus ruvB* |
| ruvBUP-R | AACTGCGTGTCAGGGCGGGTGGGCCGGATAACAGG |  |
| ruvBDown-F | ACCCGCCCTGACACGCAGTTTCG |  |
| ruvBDown-R | CTCGAGGTCGACGGTATCGATTTCGGGGGATGTGAGCAAA |  |
| recRUP-F | CTCGAGGTCGACGGTATCGATACCGTCACCGATGCCGCTGTT | Construction of the pBluescript II SK (+)-UrecRD plasmid containing the upstream and downstream homologous arms of *M. abscessus recR* |
| recRUP-R | ATCCGCACCAAGTAGTCCTGGACCGGGCCCTCAAA |  |
| recRDown-F | CAGGACTACTTGGTGCGGATGCTGC |  |
| recRDown-R | CGCTCTAGAACTAGTGGATCCTGATCGACTTCGAGGTGGAA |  |
| recCUP-F | CGCTCTAGAACTAGTGGATCCACGCCCTGAGCGCCGCAGTT | Construction of the pBluescript II SK (+)-UrecCD plasmid containing the upstream and downstream homologous arms of *M. abscessus recC* |
| recCUP-R | TCGTTTCCCCCTCGGTCTCTCGCACGCCCGCACACACCC |  |
| recCDown-F | AGAGACCGAGGGGGAAACGAA |  |
| recCDown-R | CTCGAGGTCGACGGTATCGATAGGCGCAATGCCGCCGGATAG |  |
| kuUP-F | CGCTCTAGAACTAGTGGATCCAGGCCCTTGCTTCCACTGG | Construction of the pBluescript II SK (+)-UkuD plasmid containing the upstream and downstream homologous arms of *M. abscessus ku* |
| kuUP-R | ATAGGTGTCGGTGCGTTTGTAGCGAATCCG |  |
| kuDown-F | ACAAACGCACCGACACCTATCAGTCACAGCTGC |  |
| kuDown-R | CTCGAGGTCGACGGTATCGATCCACTACTTCAGCCAAATCCGT |  |
| ligDUP-F | CTCGAGGTCGACGGTATCGATCCAGCAGCACATACGATTTG | Construction of the pBluescript II SK (+)-UligDD plasmid containing the upstream and downstream homologous arms of *M. abscessus ligD* |
| ligDUP-R | CGGTCCTTCTCGAAGAACGAC |  |
| ligDDown-F | TCGTTCTTCGAGAAGGACCGCCGCAGCAGCGGAATAGGG |  |
| ligDDown-R | CGCTCTAGAACTAGTGGATCCTTCGTCGCGTGAGCGAGCAC |  |
| recO-crRNA-F | ATCCCTGCACCGCCTCACCGTTGGTGA | Construction of the pCRZeo-recO plasmid containing a crRNA targeting *M. abscessus recO* |
| recO-crRNA-R | AGCTTCACCAACGGTGAGGCGGTGCAGGGATCT |  |
| recA-crRNA-F | ATGGGTGAACCGAACATCACGCA | Construction of the pCRZeo-recA plasmid containing a crRNA targeting *M. abscessus recA* |
| recA-crRNA-R | AGCTTGCGTGATGTTCGGTTCACCCATCT |  |
| recR-crRNA-F | ATCATTTGTTGGGTGTCGAGGCGCA | Construction of the pCRZeo-recR plasmid containing a crRNA targeting *M. abscessus recR* |
| recR-crRNA-R | AGCTTGCGCCTCGACACCCAACAAATGATCT |  |
| ruvB-crRNA-F | ATGAGCTCGTCGGGCTCGTAGAA | Construction of the pCRZeo-ruvB plasmid containing a crRNA targeting *M. abscessus ruvB* |
| ruvB-crRNA-R | AGCTTTCTACGAGCCCGACGAGCTCATCT |  |
| recC-crRNA-F | ATCGCGCCCTGGACTACACCCTGCA | Construction of the pCRZeo-recC plasmid containing a crRNA targeting *M. abscessus* *recC* |
| recC-crRNA-R | AGCTTGCAGGGTGTAGTCCAGGGCGCGATCT |  |
| ku-crRNA-F | ATCTGGTCCAACACCGGGAAGTCGA | Construction of the pCRZeo-ku plasmid containing a crRNA targeting *M. abscessus* *ku* |
| ku-crRNA-R | AGCTTCGACTTCCCGGTGTTGGACCAGATCT |  |
| ligD-crRNA-F | ATGTGGATTGGAGCCAGAACAGCA | Construction of the pCRZeo-ligD plasmid containing a crRNA targeting *M. abscessus ligD* |
| ligD-crRNA-R | AGCTTGCTGTTCTGGCTCCAATCCACATCT |  |
| czadnB-F | GGCCAAGACAATTGCGGATCCGTCAGTCGTGAATGCATAC | Construction of the complementation plasmid carrying the *M. abscessus adnB* gene |
| czadnB-R | GTTAACTACGTCGACATCGATACCAGGACAATCAGGAACA |  |
| czadnB-Mtb-F | GGCCAAGACAATTGCGGATCCATGACCCAAACCGCGGCA | Construction of the complementation plasmid carrying the *M. tuberculosis adnB* gene |
| czadnB-Mtb-R | GTTAACTACGTCGACATCGATTCAGGTGTCCGACCGCCG |  |
| czrecO-F | GGCCAAGACAATTGCGGATCCATGCGGCTGTATCGGGACC | Construction of the complementation plasmid carrying the *M. abscessus recO* gene |
| czrecO-R | GTTAACTACGTCGACATCGATCTAGCCACCGGCTATATCCTGC |  |
| czrecO-Mtb-F | GTTAACTACGTCGACATCGATGCGAACCCGCTACAGAAAC | Construction of the complementation plasmid carrying the *M. tuberculosis recO* gene |
| czrecO-Mtb-R | GGCCAAGACAATTGCGGATCCCGACGAAACGGAGAACACC |  |
| czrecA-F | GGCCAAGACAATTGCGGATCCCGACGAAACGGAGAACACC | Construction of the complementation plasmid carrying the *M. abscessus recA* gene |
| czrecA-R | GTTAACTACGTCGACATCGATGCGAACCCGCTACAGAAAC |  |
| czrecA-Mtb-F | GGCCAAGACAATTGCGGATCCATGACGCAGACCCCCGAT | Construction of the complementation plasmid carrying the *M. tuberculosis recA* gene |
| czrecA-Mtb-R | GTTAACTACGTCGACATCGATTCAGAAGTCGACGGGGGC |  |
| czrecR-F | GGCCAAGACAATTGCGGATCCGTGTTTGAGGGCCCGGTCCA | Construction of the complementation plasmid carrying the *M. abscessus recR* gene |
| czrecR-R | GTTAACTACGTCGACATCGATCGGGTCGCCGCCCCCTCTGA |  |
| czrecR-Mtb-F | GGCCAAGACAATTGCGGATCCATGTTTGAGGGACCCGTCCA | Construction of the complementation plasmid carrying the *M. tuberculosis recR* gene |
| czrecR-Mtb-R | GTTAACTACGTCGACATCGATTCAGGCGAGCACGCGGCGG |  |
| czruvB-F | GGCCAAGACAATTGCGGATCCCGCAAAACAAGCCGAGCAA | Construction of the complementation plasmid carrying the *M. abscessus ruvB* gene |
| czruvB-R | GTTAACTACGTCGACATCGATGTACAGGCGAGGGCAAGCA |  |
| czruvB-Mtb-F | GGCCAAGACAATTGCGGATCCATGACCGAGCGGTCCGAC | Construction of the complementation plasmid carrying the *M. tuberculosis ruvB* gene |
| czruvB-Mtb-R | GTTAACTACGTCGACATCGATCTACTCGAACAACCCCGGTTG |  |

^a^The *dif* core sites are underlined.
